# Mapping Global Voices for Empowering Informal Healthcare Providers to Build a Sustainable Community-Based Primary Healthcare Model in Low- and Middle-Income Countries (LMICs)

**DOI:** 10.34172/ijhpm.8859

**Published:** 2025-05-27

**Authors:** Dhiman Debsarma, Sourindra Mohan Ghosh, Bikramaditya Kumar Choudhary

**Affiliations:** ^1^Council for Social Development, New Delhi, India.; ^2^Jawaharlal Nehru University, New Delhi, India.

## Introduction

 Informal healthcare providers (IHPs) have provided primary healthcare services to millions of rural people in low- and middle-income countries (LMICs).^1^ They are popularly known by several names, such as rural unqualified health practitioners (RUHPs), often called IHPs, village doctors, unqualified practitioners, non-trained allopathy medicine providers, unlicensed providers, non‐formal providers, non-degree health providers, and quacks across various LMICs.^1^ There are 6000, 4152, 2900, 1200, 1000, 283 000, 180 000, and 1.6 million IHPs in Uganda, Kenya, Malawi, Tanzania, Zambia^2^ (in sub-Saharan African countries), Bangladesh, Pakistan,^3^ and India (1.6 million and 15 times more than qualified doctors),^4^ respectively, in South Asian Countries. In fact, they are illegal practitioners as per state laws. For example, the Clinical Establishments Act (2010), the Medical Council of India Act (1956), the Indian Penal Code (1860), and the Drugs and Cosmetics Act (1940) in India do not support the medical practices of IHPs.^4^ Such medical regulations and rules are significantly prevalent in other LMICs globally. Yet, the majority of the RUHPs/IHPs are mainly engaged in the rural areas where inadequate healthcare services and poor quality of care in the primary health centres are significant concerns. Consequently, the vast rural population in LMICs remains dissatisfied due to the inability of public services to meet their healthcare needs; as a result, they consistently seek alternative healthcare providers and frequently rely on them. In rural areas of LMICs with limited healthcare resources, IHPs often serve as the first point of contact for outpatient consultations, health services, and home-based care for multiple illnesses and health conditions, including diarrhea, fever, reproductive health issues, maternity care, and childcare.^5^

 Emphasizing on the above contextualization we can point out that a nuanced understanding of IHPs’ roles in primary healthcare is well documented. However, several scholars highlighted poor knowledge, practice gaps,^1^ poor quality of care,^6^ and poor patient safety adherence among the IHPs. Despite these, many scholars reported immense positive hope and enthusiasm for IHPs due to their potential to become an alternative community healthcare workforce in the future. However, there are no single systematic core documents on positive hope and enthusiasm regarding IHPs, rather, the literatures are found in a very scattered format in this context. We need to synthesize these documents to gain a better understanding on what are the voices and enthusiasms expressed and advocated by the public health researchers and policy-maker communities for IHPs globally. Therefore, our study tries to map the positive voices raised and the best interventional practices proposed by public healthcare researchers, agencies, and organizations over the IHPs in the global south. The current study findings could benefit the global community of public health policy-makers and states aiming to design and implement interventions (training and skill development) for IHPs in the primary healthcare landscape in LMICs.

 References for the current Viewpoint were identified through searches on PubMed and Google Search with the search terms “Informal Healthcare Providers,” “Untrained Health Workers,” “Training of informal health workers,” and “intervention” from 2000 until August 2024. Articles were also identified through searching reference lists and from the authors’ own records. Only papers, short blogs, and policy briefs published in English were selected and reviewed. The final reference list was generated based on originality and relevance to this viewpoint.

## Evidence of Global Voice Raised by Scholars, and States for IHPs

 Through their writing, many scholars and agencies advocate positive hopes and enthusiasm from the IHPs in primary healthcare service delivery in the LMICs. In this regard, we documented several positive aspects about IHPs such as their role in improving access to healthcare, reaching geographically isolated people, providing universal health coverage, promoting local-level patient-centered care, and transforming the rural healthcare landscape in the LMICs. Now a question arises why do researchers and policy-makers tend to be vocal in favour of IHPs? To the best of our knowledge, multiple sets of potential factors could encourage public health scholars’ advocates in favour of IHPs. In the broader sense, we can point out two types of major factors that could be responsible for the advocacy voice from researchers and states around IHPs. The first approach focuses on reducing and narrowing down the magnitude of the existing harmful practices and consequences of IHPs by offering educational and skill development training programs on the basics of illness, medicine, and proper medication practices. The second one is transforming IHPs into alternative community healthcare workforces through interventions (training and skill development) to address and supplement the existing shortage of formal health practitioners. There are multiple debates about both and one has a reciprocal link to the next one. However, the first one urges policy-makers to think about alternative approaches and an initial pathway toward the second one for transforming the system of IHPs.

## Global Voice for Reducing the Harmful Practice of IHPs

 In brief, focusing on the first point of view, several studies documented that IHPs have poor knowledge, practice gaps,^1^ poor quality of care,^7^ poor patient safety adherence, and late patient referral to specialist health centers are the primary concerns for policy-makers and health experts. Ahmed and Hossain’s study in Bangladesh evaluated the knowledge and practices of these practitioners, highlighting their subpar quality and emphasizing that training is essential for enhancing their competencies across multiple domains and dimensions of practice.^7^ Oyeyemi et al study in Nigeria highlighted that IHP healthcare services are sub-standard, poor in quality, and unsafe.^8^ A comprehensive review of the literature pointed out poor quality of care and unsafe clinical practices among IHPs in LMICs.^9^ Similarly, Debsarma’s study in India highlighted knowledge, attitudes, and practices about minor illnesses/diseases among IHPs are substandard.^1^ Therefore, we can point out that reducing IHPs’ harmful healthcare practices, misdiagnosis, poor treatments, and their consequences are major concerns for ensuring quality healthcare for the people in LMICs.

## Global Voice for Introducing Intervention of IHPs in Order to Reduce Their Harmful Practices

 Public health researchers and policy-makers across the globe advocates several interventions for IHPs to reduce their harmful practices and convert them into alternative human resources for filling the healthcare service delivery gaps. In this regard, preparing IHPs as alternative manpower towards the aforementioned directions many scholars noted a set of strategies such as upgrading Knowledge, Attitude and Practice, organizing training, strengthening the skills of IHPs, transforming IHP’s system, and connecting them to formal primary healthcare systems at the local level. Thapa and colleagues’ study in India noted integrating IHPs into the healthcare framework contributes to healthcare enhancement and is a step toward improving equitable healthcare access, especially in underserved areas.^6^ Considering their potential role in the healthcare landscape, the Bangladesh government and a few state governments (West Bengal, Jharkhand, Andhra Pradesh, and Assam) in India plan to upgrade and absorb IHPs as the community health workforce, particularly in the rural areas. Debsarma’s study recommended that educating these practitioners can be possible properly through systematic and continuous interventional activities.^1^ Thapa et al highlighted that structured training programmes must be required in order to upgrade such IHPs.^6^ Similarly, public health researchers argue that quacks ought to be trained to supplement the government system.^4^ Furthermore, Thapa et al suggested that clear policies are needed to guide IHP engagement in primary healthcare.^6^

 However, these studies have not documented any strategy to improve the knowledge, practices, and skills among the IHPs/RUHPs related to minor diseases.^1^ To the best of the authors’ knowledge, only Debsarma’s study has proposed blueprint strategies for upgrading the RUHPs in the state of West Bengal in India.^1^ This study suggested multistage interventions such as targeting young practitioners, allopathic and homeopathic quack, launching ubiquitous app‐based medical learning, and government‐sponsored workshops could be significant interventions to improve the level of knowledge, change positive attitudes, and adhere to standard health practice. Das and colleagues’ study highlighted that training informal providers increased correct case management rates.^10^ They suggested that multitopic medical training for IHPs may offer an effective short-term strategy to improve their healthcare quality, reduce wrong diagnoses and treatment, and promote fast patient referrals to specialist facilities.

 In addition, a recent debate emerged regarding the potential role of IHPs in the universal health coverage for primary healthcare in LMICs.^11,12^ Considering this context, Chukwuocha et al,^13^ and Christian et al,^14^ in Nigeria study suggested that there compulsory need to connect the formal primary health centre systems and informal health providers can work together in a mutually beneficial way, especially in remote and underserved regions. A study conducted by Sieverding and Beyeler suggested supporting stronger and more consistent linkages between IHPs and public health facilities is a key step towards improving health service delivery.^15^ Ponticiello et al in Uganda highlighted that introducing educational intervention for the IHPs must be required to increase HIV testing uptake.^16^ A study conducted by Tavrow et al in Kenya recommended the compulsory introduction and implementation of educational interventions aimed at improving the knowledge and practices of informal cadres involved in medical practice.^17^

 Goodman et al proposed a kind of mix of training/capacity building for quality assurance among informal providers in sub-Saharan Africa.^18^ Goodman et al proposed a comprehensive framework for executing interventional activities in the informal medical providers in Kenya advocating consecutive multi-themes, and multi-sectoral collaborations.^19^ Brieger and colleagues’ study in Africa proposed an Analytical Framework for executing IHP’s interventional activities which particularly consists of four components such as capacity interventions (workshops, training courses, peer education, and in-shop education), enabling environment (legislation and policy), demand generation (communication media and community promoters) and quality assurance (monitoring and supervision).^20^ Chalker and colleagues’ study in Vietnam proposed multi-faceted strategies and multi-component educational and capacity-building interventions that must be needed in order to upgrade the IHP’s knowledge, practice, quality of care, and good health outcomes for the population.^21^ Babar has suggested a mandatory framework of Continuing Professional Development programs for improving the clinical skills of IHPs in LMICs.^22^

 However, to establish sustainable collaboration with the formal healthcare system, it is essential to upgrade the IHPs and their healthcare systems in multiple directions and dimensions as their poor knowledge and quality of healthcare are concerns. Sudhinaraset et al suggested there is a compulsory need to collaborate and initiatives among researchers, donors, and policy-makers in introducing innovative complementary medical education intervention and their implementing and generating evidence-based results for upgrading informal healthcare systems in many directions.^23^ They have proposed educational interventions such as capacity-building training and continuing education requirements for IHPs through multi-stakeholder collaboration. Sikder advocates that interventions (training programs) must be multi-topic oriented in order to improve the quality of care ^24^. In addition, Debsarma and Choudhary reported evidence of the changing landscape in the informal healthcare system in the state of West Bengal in India with direct interventions (structural development and upgradation: skill development of IHPs, establishment of new pathology lab, recruitment of nurses or pathologist/technicians, and building and strengthening business networks, etc) introduced by formal private healthcare sectors (or IHPs themselves as they want to be upgraded into higher level healthcare system like rural health clinic).^4^ He noted that IHPs and qualified private medical practitioners have collaboratively attempted to establish a new type of quasi-healthcare model in the rural healthcare landscape. This is a kind of exceptionally new way of upgrading the informal healthcare system and IHPs driven by qualified private medical practitioners in the rural healthcare landscape. Similarly, Onwujekwe and colleagues’ study in Nigeria has suggested advocacy by policy-makers and gatekeepers in the formal health for launching complementary skill development training for IHPs and connecting them with the formal healthcare systems.^25^ Although, it is important to highlight that these research works focus on IHPs does not address how to incorporate the legality framework in the preparation and implementation process of educational interventions for IHPs in LMICs. To address medical regulations and rules, we propose that recognizing the role and contribution of IHPs in the drafting of a country’s national health policies could be a potential way forward in LMICs worldwide. This could help resolve legal issues in educational interventional activities for IHPs globally. Nevertheless, this point opens up a new dimension of future research scope that public health scholars can explore.

 Although training and skill development programs for IHPs are still in their infancy stage, they offer an opportunity to enhance the education of community health workers, thereby contributing to better population health outcomes.^1^ For example, the World Health Organization’s (WHO’s) guidelines implemented in regions like Cuba and sub-Saharan Africa have shown that providing sufficient training to community-based health workers can bring significant improvements to health systems and health outcomes of the people.^1^ Similarly, educating IHPs and RUHPs on the basics of illnesses/diseases, diagnosis, medications, and treatments could be a crucial strategy in improving their understanding of the scientific use of medicines, the principles of essential drugs, and rational drug use, which potentially might save millions of human lives in LMICs.^1^

 However, to the best of our knowledge, there is a complete absence of large-scale initiatives aimed at fostering innovative activities in intervention implementations for informal healthcare systems, which are crucial for transforming the rural healthcare landscape ensuring good health, and saving lives of the millions of people. Therefore, we make a call to international funders to kindly sanction funds support in this direction to the national agency, and non-governmental organization to design evidence-based capacity and skill development interventional programs for the informal healthcare systems in the LMICs. At last, but not least, this paper calls for international funding and support to develop evidence-based capacity-building programs for IHPs, aiming to transform rural healthcare and save lives.

## Conclusion

 Several public health researchers across the globe are optimistic and enthusiastic about the healthcare practices of the IHPs in the rural areas in LMICs. They raise their voice around IHP’s capacity-building training program. They have proposed multitopic stage and multitopic medical training programs for IHPs in the LMICs. They are very hopeful about capacity-building training to empower IHPs, believing it will build a sustainable, community-based primary healthcare model in the rural areas in LMICs. Therefore, this viewpoint makes a call to international and national funding agencies to sanction funding support to develop evidence-based capacity-building programs for IHPs, ultimately transforming rural healthcare and saving lives in LMICs.

## Ethical issues

 Not applicable.

## Conflicts of interest

 Authors declare that they have no conflicts of interest.
